# Genome Editing in Solanaceae: Harnessing CRISPR-Cas Technology for Precision Crop Improvement

**DOI:** 10.3390/plants15172715

**Published:** 2026-09-04

**Authors:** Vandana Thakur, Akshay Kumar Vats, Rahul Kumar, Anand Kumar, Shalu Vyas, Snehel Chakravarty, Kirti Bardhan, Mouli Paul, Ambika More, Vishal Johar, Vinod Kumar, Nimmy M S, R. Ravi Teja

**Affiliations:** 1 Department of Horticulture, School of Agriculture, Lovely Professional University, Phagwara 144411, Punjab, India; vandanathakur208@yahoo.com; 2 Department of Genetics and Plant Breeding, Dr Kalam Agricultural College, Bihar Agricultural University, Bhagalpur 813210, Bihar, India; akshayvts@gmail.com; 3 Faculty of Agricultural Sciences, DAV University, Jalandhar 144008, Punjab, India; rahulihbt@gmail.com; 4 Faculty of Agricultural Sciences, GLA University, Mathura 281406, Uttar Pradesh, India; anandkumar002014@gmail.com; 5 Department of Agriculture, Sant Baba Bhag Singh University, Jalandhar 144030, Punjab, India; drshaluarya@gmail.com; 6 Advance Center on Sericulture, Bihar Agricultural University, Kishanganj 855107, Bihar, India; snehel.chak@gmail.com; 7 Department of Basic Sciences and Humanities, Navsari Agricultural University, Navsari 396450, Gujarat, India; kirtivardhan@nau.in; 8 Department of Genetics and Plant Breeding, Ramakrishna Mission Vivekananda Educational and Research Institute, Narendrapur Campus, Kolkata 743145, West Bengal, India; moulipaul999@gmail.com; 9 Department of Genetics and Plant Breeding, Vasantrao Naik Marathwada Krishi Vidyapeeth, Parbhani 431402, Maharashtra, India; ambikamore26@gmail.com; 10 Department of Molecular Biology & Biotechnology, Bihar Agricultural University, Bhagalpur 813210, Bihar, India; biotech.vinod@gmail.com; 11 ICAR-National Institute of Plant Biotechnology, Indian Agricultural Research Institute, PUSA Campus, New Delhi 110012, India; nimmybiotech@gmail.com; 12 ICAR-National Academy of Agricultural Research Management, Rajendranagar, Hyderabad 500030, Telangana, India; ravitejaravi2233@gmail.com

**Keywords:** base editing, CRISPR/Cas, genome editing, nutritional biofortification, prime editing, Solanaceae crops, stress tolerance

## Abstract

Malnutrition and climate-induced stress remain major constraints to global food and nutritional security despite the yield gains of the Green Revolution. Solanaceae crops such as tomato, potato, brinjal, and pepper are key sources of vitamins, minerals, and bioactive compounds. Yet, their genetic improvement has been limited by narrow diversity and complex polygenic traits. The advent of CRISPR/Cas-mediated genome editing provides a transformative platform for precision crop improvement by enabling targeted modification of genes controlling stress tolerance, yield, and nutritional quality. In Solanaceae, CRISPR/Cas applications have successfully enhanced resistance against major pathogens (*SlMlo1*, *SlPelo*, *SlDCL2*), improved abiotic stress tolerance through editing of *SlMAPK3*, *SlCBF1*, and *SlBZR1*, and optimized fruit quality traits via modulation of *Psy1*, *CrtR-b2*, and *fiAD2/3*. Emerging innovations, such as base and prime editing, and RNP-mediated transgene-free delivery, are expanding the precision and scope of editing. However, challenges persist, including genotype-dependent transformation, low HDR efficiency, and incomplete understanding of off-target and epigenetic effects. Integrating CRISPR with omics-guided gene discovery, efficient transformation systems, and regulatory harmonization can accelerate the development of nutritionally enriched, stress-resilient, and sustainable Solanaceae varieties. This review synthesizes recent advances, identifies critical limitations, and outlines future opportunities for deploying CRISPR/Cas technology to achieve next-generation breeding and food system resilience.

## 1. Introduction

Undernutrition, overnutrition, and concealed micronutrient deficiency constitute the trifecta of malnutrition, impacting the world population more profoundly than hunger alone. Research from the World Health Organization reported that around two billion people are overweight or obese, while 462 million are underweight. Additionally, fifty-two million children are wasted, one hundred fifty-five million are stunted, and 41 million children under five years old are fat [1,2]. It has a greater impact on people with low and middle incomes worldwide because they cannot access fresh fruits and vegetables and must eat cereal-based diets that lack sufficient nutrients to meet their bodies’ needs [1,3]. On the other hand, communities with stable economies generally eat Western diets that are heavy in calories, saturated fat, sugar, and animal proteins. The result may lead to Western non-communicable degenerative diseases such as diabetes, obesity, and heart disease [2,4]. In 2016, around seventy-one percent of worldwide fatalities were attributable to non-communicable diseases, underscoring the substantial influence of diet-related disorders on health services [5]. Numerous reputable organizations, including the Food and Agriculture Organization, World Health Organization, the European Food Safety Authority, and the United States Department of Agriculture, emphasize the importance of fruit and vegetable consumption for public health [2,6,7]. The World Health Organization recommends eating at least 400 g of fruits and vegetables every day to help prevent chronic illnesses, including cancer, heart disease, and diabetes. It can also help you receive enough micronutrients, especially calcium, iron, vitamin A, zinc, and iodine. In this context, fruits and vegetables are essential for food and nutritional security because they are rich in micronutrients such as iron, calcium, zinc, iodine, potassium, folate, vitamin A, vitamin C, and dietary fiber, all of which are important for staying healthy [8]. Vegetables are frequently high in potassium, which helps keep blood pressure at a healthy level. Their nutritional makeup also helps with heart problems by lowering cholesterol levels in the blood.

Folate lowers the risk of birth defects, vitamin A protects the health of the eyes and skin, and vitamin C helps with iron absorption and dental hygiene. As a result, the global food system changed its emphasis from how much food people eat to how healthy it is, how many different kinds of food there are, and how healthy it is. This change and consumers’ awareness of how to lessen the triple burden of malnutrition have made fruits, nuts, and vegetables a top priority for them. The successful emergence of the Green Revolution, characterized by the acceptance and widespread distribution of improved varieties, significantly increased global food output. Since the beginning of the Green Revolution in 1960, by 2000, developing nations had a significant rise in agricultural yields, i.e., 208% for wheat, 109% for rice, 157% for maize, 78% for potatoes, and 36% for cassava. Notwithstanding this advancement, since 2015, the global hunger trajectory has reversed, demonstrating a gradual but consistent rise. In 2018, almost 820 million people, or nearly 11% of the global population, experienced chronic undernourishment [5,9,10]. By 2050, the global population is projected to reach over 9.9 billion, requiring increased food production to address food availability.

Simultaneously, climate change and biodiversity decline are intensifying, presenting substantial challenges to agricultural output. Plants face several biotic pressures, including bacterial, fungal, and viral diseases, and abiotic challenges such as drought, salinity, heavy metals, cold, and elevated temperatures, which therefore restrict crop output and quality. Advancements in agricultural technology have produced crops with increased yields and improved resistance to environmental stresses. Fortunately, plant breeding is a cost-efficient method to enhance the qualitative attributes of agricultural plants, hence providing additional health advantages. Conventional plant breeding techniques include the selection and hybridization of plants exhibiting desirable characteristics; however, they are limited to sexually compatible species and are a protracted process [11]. Regrettably, traditional breeding often reduces genetic diversity [12,13]. Chemical mutagenesis and radiation-induced mutation breeding generate genetic variation via point mutations. Nonetheless, they are constrained by biological and environmental safety considerations. Mutations often occur owing to the interaction between radiation and DNA, resulting in direct structural and functional modifications to DNA molecules or via indirect effects caused by free radical interactions. Nonetheless, the infrequency of advantageous mutants and challenges in forecasting their direction and kinds, coupled with a deficiency in accuracy and control, constrain its efficacy in producing desirable alleles [14]. These difficulties hinder efforts to tackle global food security issues.

Utilizing biotechnological technologies and procedures, plant breeding facilitated the fast introgression of specific genes without linkage drag in a reduced timeframe. This highlights the need for the second Green Revolution, emphasizing not only increased output but also improved nutrition and quality as primary breeding goals. These initiatives may successfully address malnutrition and chronic illnesses via integrated interdisciplinary strategies. In recent decades, several transgenic crops have been engineered by the introgression of specific genes for herbivore resistance, such as cystatin in potato and brinjal [15]. *Cry* gene in brinjal, cabbage, and okra [16,17,18], trypsin inhibitors in cauliflower [19], disease resistance in the following, such as defensin in tomato and chilli [20], glucanase in potato and brinjal [21], and chitinase in potato and tomato [22,23]. Nonetheless, the commercialization of transgenics is constrained, with notable exceptions such as the Flavr-Savr tomato, which boasts an extended shelf life, and the genetically modified papaya, which exhibits resistance to the ring spot virus. Additionally, other commercial crops incorporating the engineered BT toxin gene for insect-pest resistance constitute a significant portion of globally cultivated GM crops [24]. RNA interference (RNAi) is the most effective method, extensively utilized for the stable expression of traits including resistance to viruses, fungi, and bacteria, as well as tolerance to drought, salinity, heat, and cold, alongside enhancements in quality traits such as shelf life, plant architecture, texture, and color [25,26]. The existence of transgenes and the food safety of humans remain contentious issues [27].

The advent of molecular genome editing tools provides promising answers by facilitating precise and efficient alteration of desirable features using tailored endonucleases that may break DNA in conjunction with a specific DNA-binding domain, with or without the insertion of foreign genes. There are four primary genome editing approaches using various nuclease complexes, each with its advantages and disadvantages. Meganucleases, zinc-finger nucleases, transcription activator-like effector nucleases, and the clustered regularly interspaced short palindromic repeats with CRISPR-associated protein (CRISPR/Cas) technique. Meganucleases, ZFNs, and TALENs need intricate and expensive protein engineering, resulting in poor efficiency. Conversely, CRISPR/Cas9 provides elevated efficiency, cost-effectiveness, simplicity, multiplexing capabilities, and accessibility [28,29,30,31]. The advent of CRISPR/Cas9 significantly enhanced vegetable crop development. The tomato is the first vegetable crop to undergo CRISPR/Cas9 editing, resulting in a loss of the argonaut gene, which leads to the development of wiry or needle-shaped leaves [32,33]. Since then, the CRISPR/Cas tool has been extensively utilized in the enhancement of various vegetables, including tomato [34,35], potato [36], cassava [37], cucumber [38], cabbage [39], watermelon [40], carrot [41], lettuce [42], cauliflower [43], radish [44], and spinach [45]. The contemporary worldwide market seeks nutrient-dense, high-quality vegetables that exhibit disease resistance, possess an extended shelf life, need a shorter growing time, and necessitate less pesticide use to guarantee consumer-safe food. Consequently, substantial efforts and expenditures in research are undertaken to provide high-quality, pathogen-free seeds to producers and consumers [46].

This article encapsulates a comprehensive analysis of recent advancements in vegetable crops using CRISPR/Cas technology to enhance particular features such as nutritional value, processing quality, and resilience to biotic and abiotic challenges. Additionally, we provided insights on the use of CRISPR methodologies beyond mere editing. It also summarizes the problems and laws associated with the economic viability of CRISPR/Cas-edited vegetable plants. The discussion encompasses the nascent gene editing technology, particularly the integration of CRISPR/Cas with protoplast technology and artificial intelligence methodologies that facilitate the progression of CRISPR/Cas technology.

## 2. Review Methodology

This review was prepared using a structured narrative review approach to summarize recent advances in CRISPR/Cas-mediated genome editing for solanaceae crop improvement. A comprehensive literature search was conducted using Web of Science, PubMed, ScienceDirect, SpringerLink, and Google Scholar. Publications published between January 2013 and March 2026 were considered, while landmark studies preceding this period were included to provide the historical background of CRISPR/Cas technology. The search employed combinations of keywords and Boolean operators, including CRISPR/Cas, genome editing, Solanaceae, tomato, potato, eggplant, pepper, biotic stress, abiotic stress, nutritional quality, biofortification, base editing, prime editing, and precision breeding. Additional relevant publications were identified through cross-referencing the bibliographies of selected articles. Studies were included if they were peer-reviewed, published in English, and reported original research or review articles on CRISPR/Cas application in solanaceous crops. Conference abstracts, theses, patents, duplicate records, and studies lacking sufficient methodological information were excluded. The selected literature was critically synthesized into thematic areas, including CRISPR/Cas system, genome editing strategies, stress tolerance, quality improvement, emerging technologies, regulatory aspects, and future perspectives. As this is a structured narrative review, the emphasis was placed on critically evaluating current evidence, identifying research trends, and highlighting knowledge gaps rather than performing a formal systematic review or meta-analysis.

## 3. Site-Directed Nuclease Approaches for Crop Improvement in Vegetable Crops

Genome editing effectively remedies the constraints associated with using site-directed nucleases. This represents a significant advancement in contemporary plant breeding technology, offering effective solutions to the limitations inherent in traditional breeding and transgenic methods. SDNs are designed molecular instruments that allow precise alterations at designated loci throughout the genome. Diverse nuclease systems, such as meganucleases, zinc finger nucleases, transcription activator-like effector nucleases, and the CRISPR/Cas system, have shown significant efficacy in genome engineering applications [47]. These methods thereby enable the accurate modification of endogenous genes or targeted incorporation of foreign sequences into the genomes of recipient species, thereby improving the accuracy and predictability of genetic alterations. The prospective uses of *SDN*-mediated genome editing are many and continue to grow with continuous advancements that improve target specificity and editing precision. To enhance clarity in description and assist future regulatory evaluations of genome-edited goods, *SDN*-based alterations are classified into three primary categories: *SDN1*, *SDN2*, and *SDN3*. The *SDN1* method entails the creation of site-specific double-strand breaks, mostly repaired by the non-homologous end joining pathway, leading to random insertions or deletions (indels) at the target locus. These double-strand breaks may lead to significant chromosomal rearrangements, including extensive deletions, duplications, inversions, or translocations [48]. The *SDN2* method utilizes an external donor DNA template with specified sequence modifications that exhibit similarity to the target locus, enabling accurate nucleotide substitutions, insertions, or deletions via homologous recombination (HR)-mediated repair. The *SDN3* strategy enables the incorporation of larger DNA fragments, frequently including transgenes, into designated genomic locations through donor templates and homologous recombination repair mechanisms, thereby ensuring targeted integration and reducing the random insertion occurrences commonly linked to traditional transgenesis [49]. Site-directed nuclease (SDN)-based genome editing employs a variety of nucleases, including meganucleases (MNs), zinc finger nucleases (ZFNs), transcription activator-like effector nucleases (TALENs), and CRISPR/Cas systems, each with distinct mechanistic advantages and limitations. MNs are characterized by their small molecular size and high target specificity, enabling efficient genome manipulation with minimal cellular damage and low off-target effects. However, their structural complexity and the intricate protein–DNA recognition patterns required for customization substantially restrict their versatility and practical application [50,51]. ZFNs and TALENs represent chimeric genome editing platforms that combine a non-specific FokI endonuclease domain with customizable DNA-binding motifs derived from zinc finger proteins or transcription activator-like effectors, respectively. These modular architectures permit the recognition of nearly any target sequence of interest. Despite this flexibility, ZFNs generally exhibit reduced binding specificity relative to TALENs and MNs, which can increase the likelihood of off-target double-strand breaks (DSBs) and consequent cytotoxicity [49]. The CRISPR/Cas system, originally discovered as a prokaryotic adaptive immune mechanism against invading genetic elements such as bacteriophages, has been repurposed as a versatile and efficient genome editing tool. In 2013, Jennifer Doudna and Emmanuelle Charpentier demonstrated the programmable CRISPR/Cas9 system, which functions analogously to SDNs. Cas9, a dual-nuclease enzyme containing HNH and RuvC domains, cleaves the complementary and non-complementary DNA strands, respectively. The CRISPR/Cas9 complex comprises a CRISPR RNA (crRNA), a trans-activating crRNA (tracrRNA), and the Cas9 endonuclease. Guided by *crRNA*–*tracrRNA* duplexes, Cas9 introduces a double-stranded cut three nucleotides upstream of the protospacer adjacent motif (PAM) sequence within the target DNA [52,53,54]. The induced DSBs are repaired by either the error-prone non-homologous end joining pathway, resulting in small insertions or deletions (indels), or by homology-directed repair, which utilizes an exogenous donor template to achieve precise sequence modification but with generally lower efficiency [55]. Beyond conventional SDN-induced DSB repair, advanced precision genome editing technologies now enable single-base modifications without introducing double-strand breaks. Base editing platforms employing deaminase enzymes, such as cytosine base editors and adenine base editors, facilitate targeted cytosine-to-thymine and adenine-to-guanine conversions, respectively. Recently developed glycosylase-based guanine base editors (gGBEs) further expand this toolkit by enabling direct guanine-to-cytosine or Guanine to thymine conversions [56]. Although base editors typically achieve edits spanning one to three nucleotides, optimization of linker sequences and deaminase specificity has greatly enhanced editing precision and minimized off-target mutagenesis [57]. The prime editing system represents another major advancement, utilizing a Cas9 nickase fused to a reverse transcriptase enzyme. This approach enables precise nucleotide substitutions, insertions, or deletions without the need for double-strand breaks or donor DNA templates [58,59,60,61]. Although several CRISPR–Cas systems such as Cas12a (Cpf1), Cas13, and Cas14 have been developed, the Cas9 platform remains the most widely used in Solanaceae crops such as tomato (*Solanum lycopersicum*), potato (*Solanum tuberosum*), and pepper (*Capsicum annuum*) due to its high efficiency, simplicity, and well-established optimization protocols [62,63]. Other CRISPR–Cas systems have seen slower adoption in these crops because of limitations such as lower editing efficiency or uncertain performance in Solanaceae backgrounds, difficulties in delivering larger or more complex Cas proteins into plant cells, and a lack of optimized guide-RNA design tools and expression systems specifically tailored for Solanaceae genomes [64,65]. In addition, regeneration and transformation protocols for many Solanaceae species are already tailored for Cas9-based vectors, which makes integrating alternative systems more challenging. Limited experimental data and fewer successful reports using non-Cas9 variants in Solanaceae crops also contribute to their slow adoption compared to the well-validated Cas9 platform [65,66]. Recent research has extended genome engineering beyond single-gene modification to encompass large-scale chromosomal rearrangements, including inversions of chromosomal fragments spanning up to seventeen megabases. Such chromosomal engineering allows the suppression of meiotic recombination across entire chromosomes, thereby generating stable, heritable genomic modifications [67].

## 4. Overview of CRISPR Design Tools and Data Sources

The degree of off-target effects caused by single-guide RNAs (sgRNAs) has been thoroughly examined using several prediction and experimental methodologies, resulting in varying findings about their extent and biological relevance [68]. Many computer programs have been developed to predict off-target effects, and a few highly precise Cas9 variants have been developed to reduce inconsistencies at target sites [69]. Sequencing-based investigations have shown that many Cas9-induced off-target events are still not explained by current computational models, even with these improvements. This difference shows how complicated Cas9-DNA interactions are and makes us think about the molecular processes that control Cas9 binding specificity and off-target cleavage [70,71]. Numerous computer algorithms have been developed to detect and measure these fluctuations, offering insights into off-target prediction and assessment [72]. For example, PEM-seq analysis has been used to fully test the correctness and effectiveness of CRISPR/Cas9 editing systems. This gives useful information for choosing the best genome editing technique for certain loci [69]. This is especially essential since Cas9 works well at genomic locations with one to three mismatches compared to the sgRNA sequence [70]. CCTop is one of the most popular prediction tools. It has an easy-to-use interface and lets you change the settings to find, score, and rank possible sgRNA target sites based on their off-target potential. Researchers often use it to find high-quality target sequences in investigations of gene knockout, non-homologous end joining (NHEJ), and homology-directed repair (HDR) [73]. CROP-IT is another powerful web-based technology that uses genome-wide information, such as Cas9 editing profiles, to make it easier to forecast both binding locations and off-target cleavage events. CROP-IT improves the accuracy of Cas9 activity predictions and helps with the logical design of sgRNAs by systematically screening and rating possible off-targets. CHOPCHOP is another flexible platform that can take many types of input data and offers several ways to choose particular targets. It uses computational models to accurately forecast possible off-target editing by CRISPR/Cas9 and TALENs. It also suggests good restriction sites and primer designs for mutation validation [2,74]. So, CHOPCHOP is a great tool for quickly and accurately picking targets in a wide variety of species. The Cas-OFFinder tool can find probable off-target mutations in user-defined sequences or whole genomes quite well. Cas-OFFinder does not limit the number of mismatches and can handle different Cas9-recognized PAM variations [69]. This is different from prior RGEN prediction tools. A wide range of techniques has been used over time to determine how large and severe off-target impacts are. Nonetheless, the development of sophisticated and versatile computational instruments is crucial for attaining accurate genome editing results. These platforms should not only allow for flexible sequence changes, but they should also make it easier to study how Cas9 binds and cuts DNA in detail. Tools like CRISPR Design and CRISPR-P have made it easier to forecast off-target effects in plants, but more work is needed to make them more accurate, easier to use, and more widely available. Table 1 shows a list of some of the best design and prediction tools that are presently available to help reduce CRISPR/Cas9 off-target effects.

The available computational platforms for CRISPR guide RNA design differ considerably in their genome coverage, CRISPR nuclease compatibility, off-target prediction strategies, and suitability for plant genome editing. Among these, CRISPR-P 2.0 is specifically optimized for plant genomes and incorporates genome annotations for several crop species, including tomato (*Solanum lycopersicum* L.), making it particularly suitable for Solanaceae genome editing studies [34,77]. It supports both Cas9 and Cas12a nucleases, together with plant-specific off-target analysis, and facilitates efficient sgRNA design for genes associated with disease resistance, abiotic stress tolerance, fruit quality, and yield improvement. In contrast, CHOPCHOP v3 provides broader flexibility by supporting multiple CRISPR systems, including Cas9, Cas12, Cas13, CRISPR interference (CRISPRi) and CRISPR activation (CRISPRa), base editing, and prime editing, while accommodating diverse PAM requirements and integrating on-target efficiency and off-target scoring algorithms [82]. Consequently, CHOPCHOP is well suited for advanced genome editing applications in tomato, potato, eggplant, and pepper, particularly where alternative nuclease or precision editing technologies are employed. Cas-OFFinder differs from guide RNA design platforms by focusing on comprehensive genome-wide identification of potential off-sites using defined PAM sequences and exhaustive mismatch searches, thereby serving as an effective complementary tool for validating candidate sgRNA before experimental transformation [79]. Similarly, CCTop applies mismatch position weighting to rank candidate sgRNAs and provides details of off-target information, although it is primarily optimized for Cas9-mediated genome editing [73]. Earlier platforms, including E -CRISP and CRISPR design, contributed significantly to the development of computational guide RNA design but support fewer genomes and CRISPR modalities than recently developed platforms [75,80]. Importantly, computational predictions alone cannot fully account for genome-editing outcomes, because chromatin accessibility, guide RNA secondary structure, DNA repair pathways, and species genome architecture also influence editing efficiency and specifically [64]. Therefore, an integrated workflow combing plant specific sgRNA design using CRISPR-P 2.0 or the versatile editing capabilities of CHOPCHOP with independent genome-wide off-target validation using Cas-OFFinder is recommended for CRISPR/Cas mediated genome editing in Solanaceae crops. Such a strategy is expected to improve editing precision, minimize unintended mutations and enhance the reproducibility of genome editing experiments across economically important Solanaceae species.

## 5. CRISPR/Cas System Classification, Their Mechanistic Diversity, and Functional Architecture

The CRISPR-Cas system is an adaptive innate defense mechanism in bacteria and archaea. It has a wide range of gene composition, genomic architecture, and effector protein structure. This system is based on repeated DNA sequences (CRISPR repeats, usually 25–50 nucleotides) that are next to spacer sequences that come from invading genetic elements like plasmids or bacteriophages. These spacers help the host identify and destroy foreign nucleic acids during later infections [83,84,85]. The CRISPR-Cas defensive system works in two main steps: adaptation and maturation. During the adaptation stage, the foreign DNA fragments are added to the CRISPR locus. During the maturation stage, the CRISPR array is transcribed, and the pre-RNA is turned into mature crRNA molecules. These molecules tell the interference complex where to cut target nucleic acids [85]. CRISPR-Cas systems are primarily classified according to the composition of Cas proteins and the arrangement of effector modules. There are two main classes, six kinds, and several subtypes [85,86]. Class 1 systems (Types I, III, and IV) use crRNA-effector complexes with more than one subunit. Class 2 systems (Types II, V, and VI) use just one multidomain effector protein. Types I and III are prevalent in both archaea and bacteria, but Type IV has a primitive structure and is devoid of an adaptability module. On the other hand, the Class 2 Type II system is just for bacteria [87,88]. The Cas1 and Cas2 proteins are globally conserved and facilitate the acquisition and integration of spacers into the CRISPR array [89,90]. In Class 1 systems, the effector complex is made up of numerous Cas proteins, including Cas3, Cas5–Cas8, Cas10, and Cas11. These proteins have RNA recognition motifs called RAMPs [91]. Type I systems use Cas3, which has helicase and nuclease domains, in conjunction with Cas6e for the maturation of crRNA [92]. Type III systems have HD nucleases that are related to Cas10 and are responsible for transcription-coupled DNA cleavage. Type IV systems, on the other hand, are evolutionarily derived and do not have adaptation or interference modules, but they do have Cas5 and Cas7 homologs [93]. The Class 2 systems are easier to build, yet do a lot of different things. The Type II system (Cas9) has HNH and RuvC nuclease domains that cut DNA in two strands [94,95]. In this approach, crRNA maturation requires a tracrRNA–crRNA duplex that is broken up by RNase III and Cas9 [96]. The Type V (Cas12) effectors use just a RuvC-like domain for double-strand DNA cleavage and identify more extensive PAMs (TTTV), resulting in enhanced target selectivity [97,98]. Type VI (Cas13) effectors, which have two HEPN domains, are RNA-guided ribonucleases that target RNA transcripts [99,100,101]. Even if there are many types, it is still hard to tell the difference between subtypes in Class 2 since the domain design is the same. Newly detected systems that do not exhibit considerable resemblance to established variations are classified as unique subtypes. This classification has resulted in the discovery of three subtypes in Type II, eleven in Type V, and four in Type VI CRISPR-Cas systems [93]. Cas12a has recently evolved into a useful tool for genome editing using CRISPR. It has a unique TTTV protospacer adjacent motif (PAM) requirement and produces staggered double-stranded breaks (DSBs) with single-stranded overhangs. A comparative study of CRISPR-Cas9 and Cas12 has been done by Slaman et al. [102] in tomato cells. In tomato (*Solanum lycopersicum*), Cas12a-mediated genome editing has been thoroughly elucidated using high-throughput amplicon sequencing in protoplast systems. The *Lachnospiraceae* bacteria Cas12a (LbCas12a) had the greatest editing efficiency among the evaluated orthologs. To make it easier to use, a cloning technique based on the Golden Gate method was also designed for quick and dependable crRNA assembly. We compared LbCas12a with the more common Streptococcus pyogenes Cas9 (SpCas9) in 35 shared target loci and of several anticipated off-target sites (57 for LbCas12a and 100 for SpCas9). The results showed that LbCas12a performed as well as SpCas9 when targeting specific genes, but it caused more frequent and bigger deletions, which is useful for gene knockout applications. There was minimal off-target activity in LbCas12a; it was only found at 10 of 57 sites, usually with one or two mismatches far from the PAM. This demonstrates that the editing was quite precise [102]. The findings of Slaman et al. [102] have significant implications for CRISPR-based improvement of Solanaceae crops. The broader target provided by the TTTV Protospacer adjacent motif (PAM) of Cas12 expanded the range of editable genomic loci compared with SpCas9, thereby increasing opportunities to modify genes controlling disease resistance, abiotic stress tolerance, fruit quality, nutritional traits, and post-harvest shelf life [103]. Furthermore, the ability of Cas12a to generate staggered DNA double-strand breaks and larger deletions makes it particularly advantageous for gene knockout studies and functional genomics, facilitating the identification and validation of candidate genes involved in important agronomic traits [64]. The successful implementation of LbCas12a in tomato also demonstrates its compatibility with existing transformation and regeneration systems in this crop, suggesting its potential applicability to other Solanaceae species such as potato, eggplant, and pepper. However, further optimization of species-specific transformation protocols is required before routine breeding applications can be achieved [64,102]. Nevertheless, editing efficiency remains influenced by several biological and technical factors, including guide design, target -site accessibility, genotype, transformation efficiency, and regeneration capacity, emphasizing the importance of optimizing CRISPR delivery strategies and tissue culture systems for different Solanaceae crops [103]. Therefore, integrating Cas12 with improved transformation protocols, robust guide RNA design, and comprehensive off-target validation is expected to enhance editing precision and accelerate the development of climate-resilient, disease-resistant, and high-quality Solanaceae cultivars through precision breeding [103].

## 6. Application of CRISPR/Cas Technology in Solanaceae Crops

### Resistance to Biotic Stress in Tomato (Solanum lycopersicum L.)

**Figure 1:**
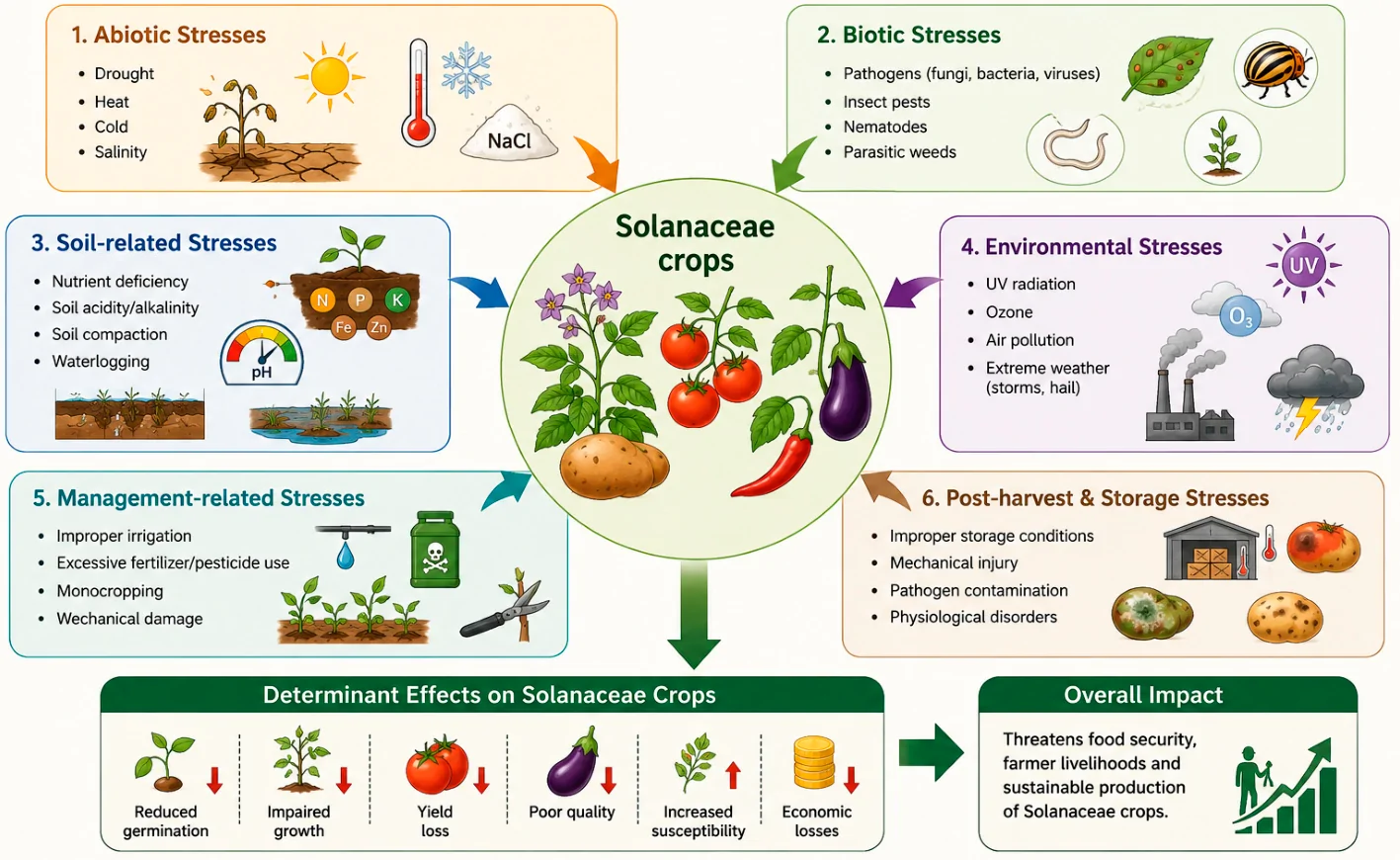
Determinant effects of different stresses on the Solanaceae crops.

**Figure 2:**
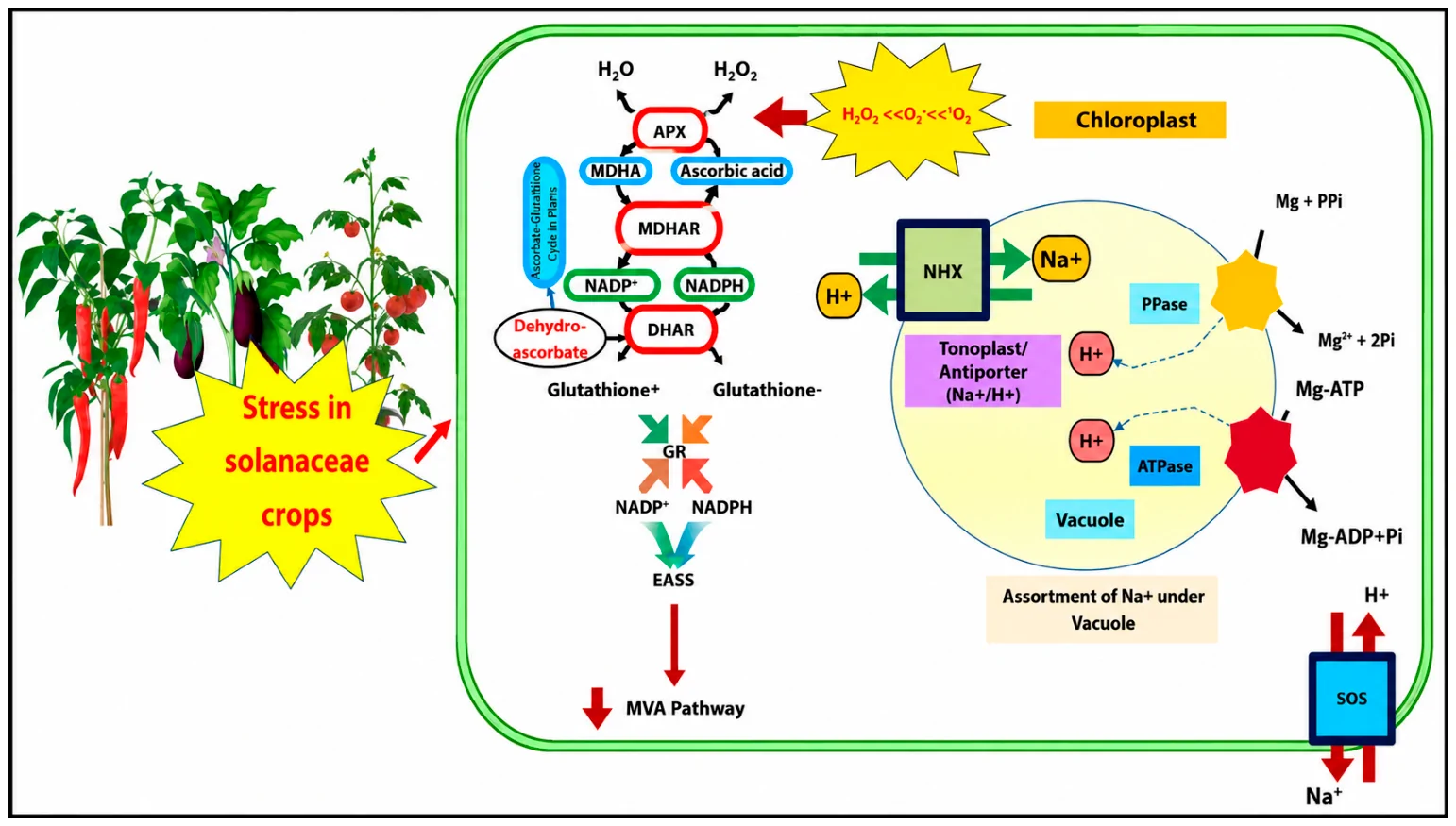
Diagram of the process of abiotic stress in solanaceous crops (e.g., abiotic stress like salinity stress in Tomato, chilli, and brinjal). Under abiotic stress like salts, reactive oxygen species promote antioxidant scavenging systems, which assist in mitigating the adverse impacts of this stress.

Various abiotic and biotic stresses considerably influence the growth, yield, and quality of Solanaceous crops (Figure 1 and Figure 2). Traditional chemical management practices have resulted in various challenges, as the continuous use of pesticides frequently leads to resistance development in pathogens and pests, diminishing chemical effectiveness and potentially fostering the emergence of new diseases [104,105]. Tomato (*Solanum lycopersicum*), a member of the Solanaceae family, is notably susceptible to various bacterial, viral, and fungal pathogens, leading to significant reductions in yield and quality globally [75]. Genetic engineering provides a viable and sustainable alternative to conventional pest management through the targeted modification of genes linked to disease susceptibility and stress tolerance. The CRISPR/Cas system, initially identified as an adaptive immune mechanism in prokaryotes, has developed into a significant tool for enhancing molecular immunity in plants against diverse pathogens, especially viruses [106]. Genomic studies have identified sixteen *Mlo* genes in tomato, with *SlMlo1* being linked to susceptibility to powdery mildew disease [107]. The *SlMlo1* gene functions as a susceptibility (S) gene that negatively regulates the pre-invasive defense response. Disruption of this gene prevents fungal penetration and enhances host innate immunity, making *SlMlo1* an attractive genome editing target for achieving durable and broad-spectrum resistance without directly targeting the pathogen. Researchers utilized CRISPR/Cas9 gene editing to create a resistant tomato cultivar named “Tomelo” by deleting a specific DNA fragment in the SlMlo1 gene, which provides lasting resistance to powdery mildew*. SlDCL2*, a crucial gene in the biogenesis of small RNAs and miRNAs necessary for antiviral defense, has been targeted with CRISPR/Cas9 to improve resistance to tobacco mosaic virus (TMV) and potato mosaic virus (PMV) [108]. Mechanically, DCL2 is a key component of the RNA interference (RNAi) pathway, where it generates virus-derived small interfering RNAs (siRNA) that direct sequence specific degradadtion of viral genomes. Manipulating this pathway strengthens the plant’s innate antiviral defense and represents an effective strategy for developing broad-spectrum virus resistance.

Subsequent research indicated that *SlJAZ2*, which encodes a protein linked to the jasmonic acid signaling pathway, is essential for modulating stomatal responses in the context of bacterial infection. The modification of *SlJAZ2* using CRISPR/Cas9 technology inhibited the reopening of stomata induced by coronatine (COR), which subsequently improved resistance to bacterial speck disease [109]. Because *SlJAZ2* acts as a negative regulator of Jasmonic acid signaling, its modification prevents pathogen-induced stomatal reopening, thereby restricting bacterial entry during the early stages of infection. This illustrates how editing regulatory genes can enhance natural immune responses without introducing foreign resistance genes.

Successful gene-editing efforts include the knockout of *SlMYC2*, which enhanced tomato resistance to *Botrytis cinerea* via improved methyl jasmonate (MeJA) responsiveness, and the disruption of *Solyc08g075770*, which provided tolerance to Fusarium wilt [110]. As a central transcriptional regulator of jasmonates signaling, *SlMYC2* coordinates multiple defense-related genes involved in pathogen perception and stress adaptation. Therefore, precise modification of this regulatory network offers opportunities to enhance resistance while maintaining normal plant development.

CRISPR/Cas9-mediated editing of the *PMR4* gene decreased susceptibility to powdery mildew, whereas *SlPelo* knockout mutants limited systemic virus movement and reduced accumulation of tomato yellow leaf curl virus (TYLCV). Additionally, *SlMlo1* mutant lines demonstrated full tolerance to powdery mildew [111,112]. The enhanced resistance observed in *PMR4* mutants is associated with altered callose deposition and reinforcement of cell wall barriers that restrict fungal invasion, whereas disruption of *SlPelo*, a gene involved in translation quality control, interferes with viral replication and long-distance movement. These studies demonstrate that CRISPR/Cas technology can target multiple host pathways to achieve durable resistance against diverse class of pathogens. Tashkandi et al. [112] demonstrated the effectiveness of Cas9-single gRNA constructs targeting the TYLCV replicase and coat protein genes, leading to a notable decrease in viral DNA accumulation in edited tomato plants (Table 2). Danilo et al. [113] documented the inaugural occurrence of transgene-free, homology-directed repair (HDR)-mediated genome editing in tomato via Agrobacterium-based CRISPR/Cas9 delivery, attaining a success rate of around 4.9%, thereby underscoring its potential for future crop enhancement initiatives. The successful implementation of transgene-free HDR editing represents an important milestone for tomato breeding because it enables precise allele replacement while minimizing regulatory concerns associated with stable transgene integration, thereby facilitating the development of improved cultivars with greater public acceptance.

**Figure 3:**
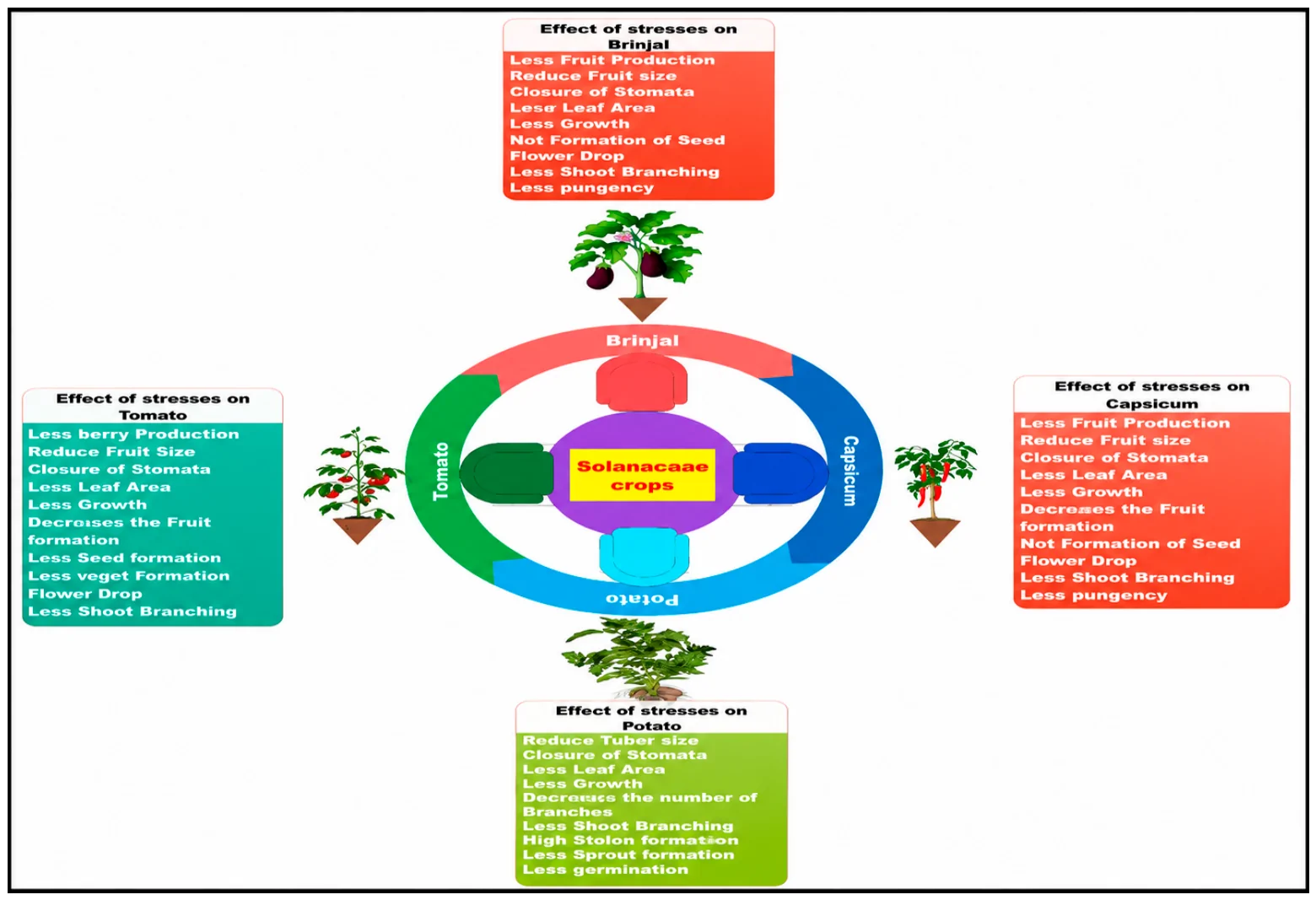
Various potential approaches for enhancing the genetic makeup of solanaceous crops.

In a comparative evaluation of PCR and CRISPR/Cas12a-based detection techniques for Tomato leaf curl Karnataka virus (ToLCKV; GenBank Accession No. PP763439.1), whole-genome sequencing was utilized to create specific guide RNAs aimed at the coat protein (CP) and replication-associated protein (Rep) genes. The produced and purified Cas12a protein (about 187 kDa) was combined with these guide RNAs to form ribonucleoprotein (RNP) complexes. Three of the four complexes tested, CPgRNA-1, CPgRNA-2, and REPgRNA-1, were ten times more sensitive than regular PCR (1.0 ng). Field validation with CPgRNA-1 and CPgRNA-2 further substantiated its diagnostic precision for ToLCKV detection in naturally infected tomato specimens. These results show that CRISPR/Cas12a-based tests are more sensitive and can be used in the field, making them the next generation of molecular tools for real-time monitoring and early detection of plant viral infections [114]. The high sensitivity of Cas12a-based diagnostics is attributed to its collateral trans cleavage activity following target recognition, enabling rapid signal amplification without sophisticated laboratory infrastructure. Such portable diagnostic platforms could support early disease surveillance and facilitate timely management decisions in tomato production systems. Tomato production faces significant limitations due to biotic stresses, including bacterial wilt, Fusarium wilt, and viral diseases such as TMV and TYLCV, which result in substantial yield and economic losses. Overreliance on chemical pesticides elevates production costs and presents considerable risks to human health and the environment. CRISPR/Cas9-mediated genome editing serves as a precise and efficient method for the development of disease-resistant tomato cultivars, while also reducing environmental risks (Figure 3). Collectively, these studies demonstrate that CRISPR/Cas-mediated improvement of tomato disease resistance is driven by the precise manipulation of susceptibility genes and key regulators of immune signalling rather than by simply introducing resistance traits. Targeting host genes that control pathogen recognition, RNA-silencing pathways, hormone signalling, and cell wall reinforcement provides a mechanistically informed strategy for developing broad-spectrum and durable resistance. Future breeding programmes should integrate multiplex genome editing with optimized transformation systems to simultaneously improve disease resistance, agronomic performance, and environmental sustainability in Solanaceae crops.

**Table 2:** Enhancement of biotic and abiotic stress resistance using CRISPR/CAS-9 technology in tomato.

Targeted Traits	Targeted Genes	Genotypes	Transformation System	CRISPR/Cas System	Knock in/Knockout	References
Drought tolerance	*SlMAPK3*	*Ailsa Craig*	*Agrobacterium*	*Cas9*	Knockout	[115]
Fusarium wilt tolerance	*Solyc08g075770*	*76R*, *rmc*	*Agrobacterium*	*Cas9*	Knockout	[110]
Chilling tolerance	*SlCBF1*	*Ailsa Craig*	*Agrobacterium tumefaciens (EHA105 strain)*	*Cas9*	Knockout	[116]
Tomato yellow leaf curl virus	*CP or Rep*	*Moneymaker*	*Agrobacterium*	*Cas9*	Knockout	[112]
Bacterial speck	*SIJAZ2*	*DC3000*	*Agrobacterium tumefaciens mediated*	*Cas9*	Knockout	[109]
*Botrytis cinerea*	*SIMYC2*	*Ailsa Craig*	*Agrobacterium*	*Cas9*	Knockout	[117]
Tomato yellow leaf curl virus	*rgsCaM*	*Moneymaker*	*Agrobacterium*	*Cas9*	Knockout	[118]
Powdery mildew resistance	*PMR4*	*Moneymaker*	*Agrobacterium*	*Cas9*	Knockout	[119]
PVY, CMV	elF4EI	*S8 (Inbred line)*	*Agrobacterium*	*Cas9*	Knockout	[120]
Downy mildew	*DMR6-1& 2*	*FL8000*	*Agrobacterium*	*Cas9*	Knockout	[121]
Pest resistance & jasmonic acid regulation	*SlMYC1*	*Micro-Tom*	*Agrobacterium (GV3101)*	*Cas9*	Knockout	[122]
Tomato yellow leaf curl virus & Powdry mildew	*SlMlo1*	*BN-86*	*Agrobacterium*	*Cas9*	Knockout	[111]
*Resistance for Phellpanche aegyptaca*	*MAX1*	*-*	*Agrobacterium tumefaciens-mediated strain EHA105*	*Cas9*	Knockout	[123]
*Resistance to P. syringae*, *P. capsici and Xanthomonas* spp.	*SlDMR6*	*Fla.8000*	*Agrobacterium*	*Cas9*	Mutagenesis	[121]
***Cas12/Cas13/Cas14***						
*Tomato Brown Rugose Fruit virus, Tobamoviruses Tomato mosaic virus*	*ORF1 region*	*Moneymaker*	*Diagnostic in nature*	*Cas12a*	Detection tool	[124]
*Bacterial Canker disease*	*SlAL2*	*Micro-Tom*	*Biolistic (microprojectile bombardment) via somatic embryogenesis*	*dCas12a*	Epigenetic activation	[125]
*Tomato leaf curl Karnatka Virus*	*CP & Rep*	*Arka Vikas*	*Pre-amplification*	*Cas12a*	Detection assay	[114]
*Tomato rugose fruit virus*	*CP gene (coat protein gene)*	*-*	*Diagnostic in nature*	*Cas12a & Cas9*	Diagnostic detection	[126]
*Geminiviruses*	*-*	*-*	*AI-based Microfluidic platform*	*Cas14a*	Diagnostic detection	[127]
*Tomato Brown rugose fruit virus*	*CP gene*	*-*	*Bacterial cloning*	*AI & Cas13a*	Diagnostic assay	[128]
*Tomato leaf curl virus*	*-*	*-*	Diagnostic assay (SgRNA)	*Cas12a (CHOP-CHOP2.0)*	Diagnostic assay	[114]
***Abiotic stress***						
Salinity	*HKT1;2, RAD51/54*	*Hongkwang*	*Agrobacterium*	*Cas12a/LbCpf1*	*Geminivirus replicon*	[129]
Salt stress tolerance	*SlHyPRP1*	*Hongkwang*	*Agrobacterium*	*Cas9*	Knockout	[130]
Water deficit	*SlARF4*	*-*	*Agrobacterium*	*Cas9*	Knockout	[131]
Multiple stresses	*GRXS14/15/16/17*	*Rubion*	*Agrobacterium*	*Cas9*	Knockout	[132]
*Heat*	*CPK28, APX2*	*Condine Red*	*Agrobacterium*	*Cas9*	Knockout	[133]
*Salinity*	*HAK20, SOS1*	*S. lycopersicum, Solanum pimpinellifolium*	*Agrobacterium*	*Cas9*	Knockout	[134]
*Herbicide resistance*	*PDS, ALS, EPSPS*	*Micro-Tom*	*Agrobacterium*	*Cas9*	Knockin	[135]
*Heat stress tolerance*	*HSPs, DREB1 & NHX1*	*-*	*-*	*Cas9*	Knock out	[136]
*Salt stress tolerance*	*PUT2, HB52*	*-*	*Agrobacterium tumefaciens mediated*	*Cas9*	Knock out and overexpression	[137]
*Tomato trichomes*	*SlHDZIV3 &SlHDZIV9*	*-*	*DNA based*	*Cas9*	Knock out	[138]
***Cas12***						
*PAM relaxed and temperature-tolerant*	*SlGGP1, SlMYBATV, SlCYCB*	*-*	*Agrobacterium*	*Mb3Cas12a*	Knock out	[139]

## 7. Abiotic Stress Tolerance in Tomato

**Figure 4:**
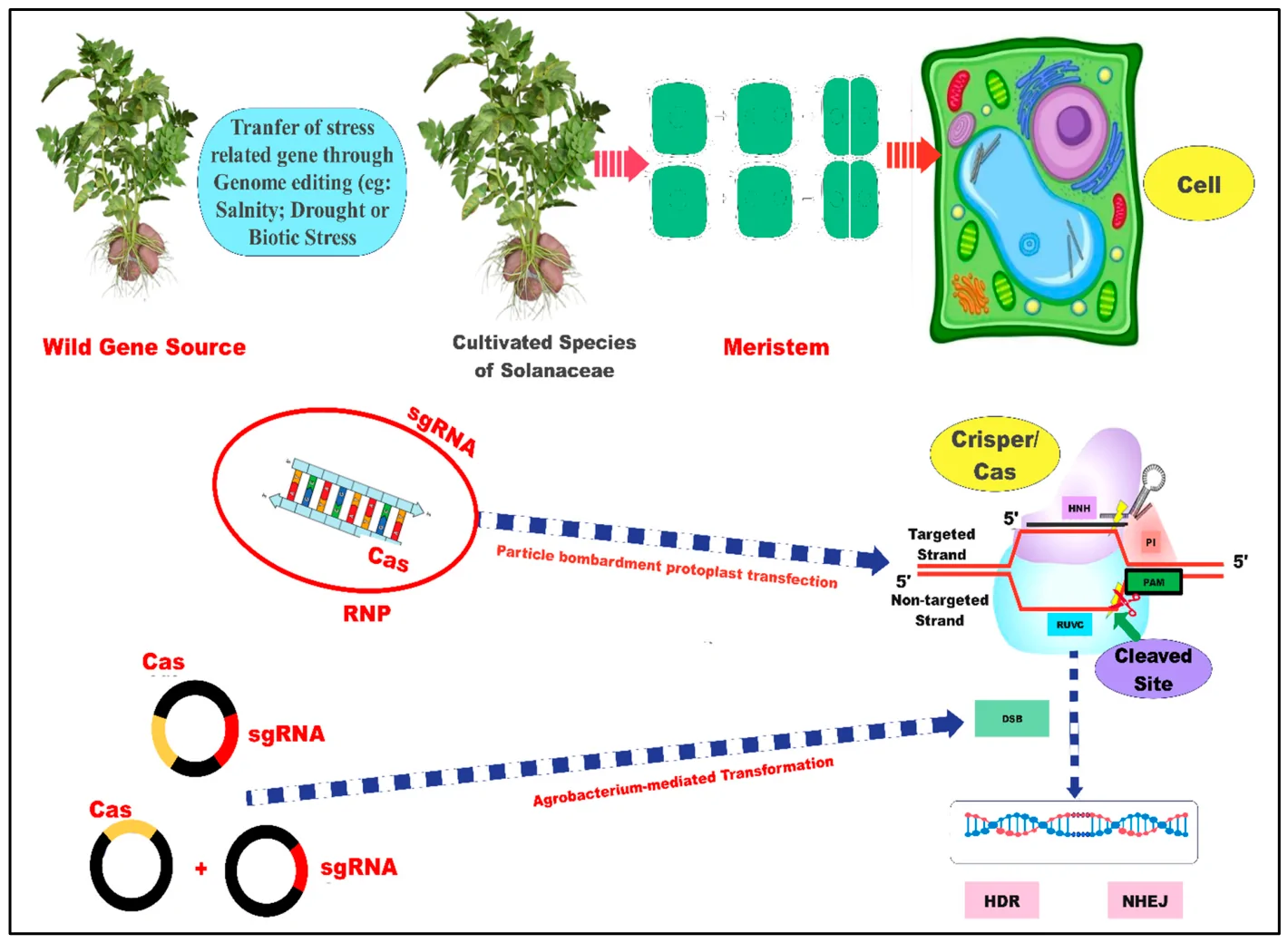
Schematic model for the transfer of stress traits to plants through the utilization of genome editing technology.

Various abiotic stresses negatively impact the growth and productivity of tomato (*Solanum lycopersicum*), with cold stress and salinity being the most harmful (Figure 1 and Figure 2). Low-temperature stress markedly diminishes yield and fruit quality in tomato crops. The SlCBF1 (C-repeat Binding Factor-1) mutant lines are recognized as significant regulators in cold stress adaptation, acting as transcriptional activators that improve tolerance to low temperatures [140,141]. Additionally, the silencing of the *SlMAPK3* gene through the CRISPR/Cas9 system led to significant phenotypic abnormalities, such as wilting, cell membrane damage, and increased hydrogen peroxide accumulation. This suggests that *SlMAPK3* is crucial for sustaining drought tolerance in tomato plants [115]. *BZR1* (Brassinazole Resistant-1) serves as a central regulator in brassinosteroid-mediated stress responses, playing a crucial role in plant growth and acclimatization to heat stress. Apoplastic hydrogen peroxide (H_2_O_2_) triggers signalling pathways that activate antioxidant enzymes, which scavenge reactive oxygen species (ROS) and improve tolerance to abiotic stresses. The impairment of *SlBZR1* function correlates with diminished expression of the *RBOH1* gene, reduced apoplastic H_2_O_2_ production, and compromised thermotolerance [142]. The *SlNPR1* gene regulates plant defense mechanisms against pathogens. CRISPR/Cas9-mediated slnpr1 mutants demonstrated enhanced tolerance to water deficit conditions [116]. Various abiotic factors, including temperature extremes, water scarcity, and mineral imbalances, directly or indirectly affect tomato yield, quality, and stress resilience [143]. The CRISPR/Cas genome editing system provides a precise method for modulating site-specific genes associated with stress responses, thereby improving abiotic stress tolerance in tomato crops. Table 2 summarizes several examples of gene additions and deletions that confer stress tolerance, while Figure 4 provides illustrations of these instances.

### 7.1. Enhancement of Tomato Fruit Quality and Other Aspects

Tomato has been widely employed as a model organism for quality improvement via CRISPR/Cas9-mediated genome editing. Various biochemical traits, including anthocyanins and carotenoids, hold considerable nutritional and physiological significance [142,144]. Zhi et al. [142] showed that the biosynthesis of anthocyanins in tomato is controlled by specific genes in the hypocotyl and cotyledon, with *SlAN2* mutants displaying pigment levels comparable to those of ‘Indigo Rose’. *CrtR-b2* and *Psy1* genes, linked to carotenoid biosynthesis, were successfully targeted using CRISPR/Cas9, demonstrating its efficacy in producing heritable mutations [144]. Nonaka et al. [145] demonstrated an increase in g-aminobutyric acid (GABA) accumulation and fruit size through the deletion of the auto-inhibitory domains of *SIGAD2* and *SIGAD3*. Genome editing led to a 5.1-fold increase in lycopene content and facilitated the induction of male sterility through *SIMS10* gene modification [146]. In potato, the insertion of a 533 bp fragment into the promoter region of the *Sli* gene resulted in decreased self-pollen rejection [147]. Additionally, the knockout of the S-RNase gene led to the development of self-compatible diploid lines that inherited this trait in the T1 generation [148]. CRISPR/Cas9 has been utilized for pest resistance, with mutagenesis of the vest gene in *Leptinotarsa decemlineata* leading to wingless phenotypes. The targeted knockout of susceptibility genes (*StDMR6-1*, *StDND1*, *StCHL1*) improved resistance to late blight [149]. Modifications to the coilin gene enhanced tolerance to PVY, drought, and salinity, whereas alterations in the *StCDF1-StFLORE* region influenced water balance and growth. While CRISPR/Cas9 (SaCas9) has facilitated accurate nucleotide substitutions in tetraploid potatoes [81], the elimination of exogenous sequences continues to pose difficulties in vegetatively propagated crops. CRISPR/Cas13a-based editing effectively conferred resistance to PVY in transgenic potato lines. CRISPR/Cas-based genome editing serves as an effective method for enhancing fruit quality, stress tolerance, and reproductive compatibility in Solanaceous crops (Table 3).

#### 7.1.1. Biotic Stress Resistance and Self-Incompatibility Mechanism in Potato (*Solanum tuberosum* L.)

RNA viruses are one of the major phytopathogens that cause severe damage to crops. These viruses are obligate intracellular pathogens with finely tuned evasion mechanisms, which may result in viral non-responsiveness to the preponderance of prophylactic treatments. They acquire the ability to prevail over the demands of the host defense. So, CRISPR/Cas9 is the way that assures host-mediated or pathogen-derived resistance towards viruses (Table 4). Besides this, the CRISPR/Cas13a technique is an alternative means to solve the PVY (potato virus Y) problem in various crops [105]. The self-compatible inbred potato had been developed through introgression of the *Sli* gene (S-locus inhibitor) keen on diploid germplasm that permits the generation of large number of self-fertilized seeds. Two vice versa performing vectors, i.e., an expression vector and CRISPR/Cas9 vectors, were used to convert self-compatible to incompatible and self-incompatible to self-compatible, respectively. The S-locus inhibitor gene-encoded F-box protein exclusively exhibited its effects in the pollen of self-compatible plants (Table 4).

#### 7.1.2. Quality Improvement in Potatoes via CRISPR/Cas9

CRISPR/Cas reagents employing two sgRNA strategies were utilized to target the *StALS-1* (acetolactate synthase-1) gene, resulting in the induction of specific mutations in potato calli. This gene is involved in the preservation of genomic stability in diploid and tetraploid potato varieties. Targeted mutations were primarily identified in diploid backgrounds, with only two specific mutation types noted at a single *ALS* locus. In tetraploids, four out of five events resulted in a single mutation type. The targeted mutations exhibited heritability across both ploidy levels, demonstrating a transmission rate of 87–100%. The knockout of the GBSS (granule-bound starch synthase) gene effectively modified starch quality [191]. Later research utilized CRISPR/Cas9 RNPs to target *GBSS (EC 2.4.1.242*), resulting in a complete knockout across four alleles and yielding 2–3% regenerated shoots [192]. The application of dMac3-installed Cas9 significantly improved mutagenesis efficiency; however, a decrease in amylose content was noted [193]. The mutation of the *St19DOX* gene using gRNAs delivered via *pMgP237* vectors in hairy root cultures resulted in glycoalkaloid-free lines, thereby confirming the successful disruption of SGA biosynthesis [194]. CRISPR/Cas9-mediated editing of the *StPPO-2* gene resulted in a 69% reduction in enzymatic activity and a 73% reduction in polyphenol oxidase (PPO) activity [195]. Additionally, mutations in the *SBE3* gene altered starch characteristics similar to those observed in BE1 rice mutants [196], while editing of the *StSSR2* gene resulted in reduced mediated gene editing provides a precise method for enhancing the nutritional quality of potatoes while reducing anti-nutritional factors and related health risks (Table 5).

## 8. Effect of CRISPR/Cas9 Technology on Various Aspects of Brinjal (*Solanum Melongena* L.)

The berries of *Solanum melongena* L. contain a high concentration of phenolic compounds, primarily chlorogenic acid (5-O-caffeoylquinic acid) [203]. A positive correlation has been observed in eggplant fruit flesh between the concentration of phenolics, specifically chlorogenic acid, and the degree of tissue browning. Other morphological and physiological parameters may also affect the browning phenomenon [204]. The commercial selection of cultivars with reduced internal browning has unintentionally resulted in the propagation of genotypes that have lower phenolic content. Six genes in eggplant are identified as encoding polyphenol oxidases (PPOs). According to sequence homology and organ-specific expression profiles, these PPOs are categorized into two primary clades: Clade A and Clade B. Clade A genes are mainly expressed in the roots, whereas Clade B genes are primarily linked to defense-related responses [205]. The classification was subsequently broadened to encompass PPOs from additional members of the Solanaceae family [204]. The genetic enhancement of brinjal via molecular techniques has been constrained until recently. The initial successful implementation of CRISPR/Cas9-mediated gene editing in eggplant was documented by Maioli et al. [206], who employed high-quality genome sequencing to identify ten *SmelPPO* genes (*SmelPPO1*–*10*). Targeted mutagenesis of *SmelPPO4*, *SmelPPO5*, and *SmelPPO6* was accomplished utilizing the CRISPR/Cas9 system. The edited in vitro-regenerated plantlets were confirmed through Illumina deep sequencing of amplicons from the target regions. The induced mutations were consistently inherited in both T_1_ and T_2_ progenies, accompanied by a reduction in PPO activity and a subsequent suppression of fruit flesh browning. Karuppannasamy et al. [207] recently reported the inaugural successful application of the CRISPR/Cas9 ribonucleoprotein (RNP) complex to edit the Tryptophan 2,3-dioxygenase gene, which regulates eye pigmentation, in the brinjal shoot and fruit borer (*Leucinodes orbonalis*). The mutant moths of both sexes displayed reddish-brown eye coloration, confirming the effectiveness of RNP-based genome editing in this species [207]. This advancement establishes a new framework for studying sex determination and spermatogenesis, while also presenting a potential precision-based strategy for pest management in eggplant cultivation. A few of the examples have been listed in Table 6.

### 8.1. Capsicum (Capsicum annuum L.) Attributes Improvement via CRISPR/Cas9 Technology

The research utilized bulked segregant analysis (BSA) to identify genetic markers associated with the cmr2 gene, which confers resistance to Cucumber mosaic virus (CMV) in Capsicum species. The analysis identified one marker, CMV AFLP, positioned 16 cM from cmr2. Additionally, *BSA-Affymetrix* (*BSA-Affy*) analysis revealed a single-nucleotide polymorphism (SNP) marker, *Affy4*, located 2.3 cM from cmr2 on chromosome 8. The screening of a pepper germplasm collection consisting of 4197 accessions identified alternative sources of *CMV-P1* resistance, with multiple accessions demonstrating resistance levels similar to the resistant cultivar ‘Lam32’. Inheritance and allelism tests validated the presence of the cmr2 gene in all resistant accessions. Genetic and molecular analyses determined that cmr2 is a recessive gene that provides significant tolerance to the virulent *CMV-P1* strain, which had previously bypassed the resistance conferred by Cmr1. This investigation primarily utilized two cultivars: the hot chili cultivar CM334 and Capsicum cv. Dempsey. Experimental results demonstrated that Agrobacterium tumefaciens strain GV3101 elicited the highest callus formation in cv. Dempsey, whereas all three Agrobacterium strains assessed yielded similar callus induction rates in CM334. Optimal concentrations of phosphinothricin (PPT) for the selection of the CRISPR/Cas binary vector (*pBAtC*) were established as 1 mg/L for CM334 and 5 mg/L for Dempsey [212]. Subsequent research utilized PEG-mediated transfection to introduce CRISPR/Cas9 or Cpf1 ribonucleoprotein (RNP) complexes into isolated protoplasts of CM334 and Dempsey [213]. Deep sequencing indicated that the editing efficiency of the *CaMLO2* gene differed among cultivars based on the RNP complex employed. Homozygous, T-DNA-free mutant lines were generated using a CRISPR/Cas9 system that targeted the *CaERF28* gene, leading to increased anthracnose resistance and upregulation of defense-related genes [214]. A limited number of successful genome-editing studies in Capsicum species have been reported, primarily concentrating on DNA-free editing methods and disease resistance (Table 7). The application of CRISPR/Cas9 technology offers a substantial opportunity to address current research gaps and to promote functional genomics and molecular breeding aimed at enhancing resistance and traits in *Capsicum* spp.

### 8.2. Bottleneck of Utilizing CRISPR/Cas9 Technology in Solanaceous Crops

Despite remarkable progress, the application of CRISPR/Cas9 in Solanaceae crops such as tomato, potato, pepper, and eggplant remains hindered by several critical bottlenecks. Major limitations include genotype-dependent transformation and regeneration recalcitrance, high genome complexity and polyploidy, low efficiency of homology-directed repair (HDR), and restricted delivery of editing components to germline or meristematic tissues, often leading to chimerism and somatic mosaicism. Off-target mutations, chromosomal rearrangements, and somaclonal variation further compromise genome stability, while the polygenic and environmentally influenced nature of key agronomic traits complicates genotype–phenotype correlation. Regulatory uncertainty and limited public acceptance also constrain field-level deployment. Moreover, organelle genome editing in Solanaceae remains underexplored due to RNA and Cas9 import barriers. Emerging research gaps include limited progress in epigenome editing, transgene-free genome editing systems, and multiplex or network-level gene targeting. Optimization of base and prime editing tools, integration of multi-omics for functional validation, understanding chromatin accessibility affecting editing precision, and long-term inheritance stability of edited traits also represent key challenges that need to be addressed for efficient and precise genome engineering in Solanaceae crops. In addition to these technical limitations, several translational bottlenecks continue to impede the widespread adoption of CRISPR/Cas technology in Solanaceae crops. Genotype-dependent transformation and regeneration remain major constraints, as efficient editing protocols developed for model cultivars are often not directly applicable to elite breeding lines. Commercial deployment is further complicated by differences in regulatory frameworks among countries, creating uncertainty in the approval and acceptance of genome-edited crops. Moreover, breeding priorities vary among Solanaceae species, requiring species-specific editing strategies to target traits such as disease resistance, abiotic stress tolerance, fruit quality, and yield. Addressing these challenges through improved transformation platforms and integration with complementary breeding technologies, including speed breeding, genomic selection, and multi-omics approaches, will be critical for translating laboratory advances into commercially viable cultivars.

## 9. Future Prospects

Vegetable crop improvement encompasses multiple breeding objectives, including enhanced yield potential, tolerance or resistance to biotic and abiotic stresses, extended postharvest shelf life, improved processing and nutritional quality, and superior aesthetic attributes. In Solanaceae crops, particularly tomato (*Solanum lycopersicum*), potato (*S. tuberosum*), capsicum (*Capsicum annuum*), and eggplant (*S. melongena*), many of these traits are polygenic and governed by complex gene networks, posing significant challenges to conventional breeding [223]. The advent of CRISPR/Cas genome-editing systems has revolutionized Solanaceae improvement by enabling precise and multiplex modification of target genes associated with desirable agronomic and quality traits. This technology facilitates targeted mutagenesis, gene knockouts, and allele replacement with high efficiency, thereby accelerating trait introgression compared to traditional mutagenesis or transgenic approaches [224]. In recent years, CRISPR/Cas9-mediated viral resistance has been successfully demonstrated in tomato and pepper by disrupting host susceptibility (S) genes or directly targeting viral genomes using sgRNAs complementary to viral DNA or RNA. Similarly, genome editing of metabolic genes such as *SlMYB12*, *SlAN2*, and *SlDFR* has been employed to modulate the biosynthesis of health-promoting secondary metabolites, including anthocyanins and flavonoids [142,225]. In potato, CRISPR/Cas-based knockout of *StGBSS* and *StVInv* genes has improved starch quality and reduced cold-induced sweetening, respectively, demonstrating its utility in tuber quality enhancement [191,226]. The availability of high-quality reference genomes and transcriptome data for major Solanaceae species has further advanced precise editing, enabling functional validation of candidate genes associated with yield, stress adaptation, and fruit development [227]. However, genome-editing efficiency still varies among genotypes due to transformation and regeneration bottlenecks, especially in commercial cultivars that are recalcitrant to *Agrobacterium*-mediated transformation. Moreover, incomplete genomic information for many wild Solanaceae relatives limits target-site prediction and off-target assessment. Although CRISPR/Cas-based editing is more precise and predictable than conventional mutagenesis, its success relies on optimized regeneration protocols and robust regulatory frameworks to ensure biosafety and acceptance. Presently, regulatory ambiguity persists in distinguishing gene-edited crops from genetically modified organisms (GMOs), hindering their commercial deployment [228,229]. Nonetheless, the ongoing refinement of delivery systems, base and prime editing platforms, and transgene-free editing methods positions CRISPR/Cas technology as a transformative tool for sustainable genetic improvement of Solanaceae crops in the near future. Future vegetable breeding should not only focus on improving yield, quality, and stress tolerance but also address the demands of modern production systems. As greenhouse cultivation, automation, artificial intelligence, robotics, and digital farming become more widespread, CRISPR/Cas can be used to develop cultivars with traits such as compact growth, uniform fruit development, and longer shelf life that are better suited for automated cultivation and harvesting. Combining CRISPR/Cas with AI-assisted breeding and high-throughput phenotyping can further speed the development of efficient and climate-resilient vegetable varieties [230].

## 10. Conclusions

The CRISPR/Cas system represents a transformative advancement in the molecular breeding of Solanaceae crops, offering unprecedented precision, efficiency, and versatility in targeted genome manipulation. In crops such as tomato and potato, this technology has already demonstrated significant success in improving yield, fruit quality, and resistance to biotic and abiotic stresses. However, its application across the broader Solanaceae family, including Capsicum and brinjal, remains comparatively limited due to crop-specific regeneration barriers, genotype dependency, and incomplete genomic resources. Despite these constraints, continuous refinement of high-fidelity Cas variants, base and prime editing systems, and efficient delivery platforms promises to overcome existing limitations. Bridging the gap between laboratory-scale editing and field-level implementation remains a critical challenge. Comprehensive field evaluation of CRISPR-edited lines, coupled with the establishment of shared databases for edited crop varieties, can facilitate regulatory clarity, biosafety monitoring, and public acceptance. Moreover, integration of CRISPR-based approaches with multi-omics, speed breeding, and AI-driven predictive tools could accelerate the development of resilient and high-performing Solanaceae cultivars. Future research should focus on developing genotype-independent transformation systems, transgene-free editing approaches, and strategies for editing complex and polyploid genomes such as those of potato. Overall, the CRISPR/Cas platform holds immense promise to revolutionize Solanaceae crop improvement by enabling precise, rapid, and sustainable genetic enhancement. With ongoing technological innovations, regulatory harmonization, and societal engagement, CRISPR/Cas-based genome editing is poised to play a pivotal role in ensuring food and nutritional security through next-generation Solanaceae breeding programs.

## Figures and Tables

**Table 1:** Tools to design the CRISPR/Cas system.

Tools	Features	References
CRISPR Design	Designing sgRNA, forecasting off-site targets, and restricted numbers of genome references	[75]
CRISPRseek	Target-specific sgRNA (forecast of off-site target)	[76]
CRISPR-P	sgRNA design tools (plants and forecast of off-site target)	[77]
CRISPR-PLANT	CRISPR/Cas9-sgRNAs (plant)	[78]
Cas-OFFinder	Multiple features, target off-site next to numerous genome references, designing of sgRNA	[79]
E-CRISP	Multiple features, designing and construction of CRISPR, and off-site forecast of restricted genome numbers	[80]
CROP-IT	CRISPR/Cas9 (target sites-input sequence & off-site-targeting prediction)	[81]
CCTop	sgRNA-target selection &off-target forecast	[73]
CHOPCHOP	Multiple features, Targeted sites cast-off for CRISPR/TALENs systems and target off-site next to numerous genome references	[82]

**Table 3:** Enhancement of quality traits and other aspects using CRISPR/CAS-9 technology in tomato.

Targeted Traits	Targeted Genes	Genotypes	Transformation System	CRISPR/Cas System	Knock in/Knockout	Reference
Fruit ripening	*RIN*	*Alisa Craig*	*Agrobacterium tumefaciens mediated*	*Cas9*	*Knockout*	[150]
Generating stable & heritable modifications	*SlPDS* & *SlPIF4*	*Micro-Tom*	*Agrobacterium*	*Cas9*	*Knockout*	[151]
GABA accumulation	*SlGAD2* & *SlGAD3*	*Micro-Tom*	*Agrobacterium*	*Cas9*	*Knockout*	[145]
Parthenocarpic fruit	*SlIAA9*	*Alisa Craig, Micro-Tom*	*Agrobacterium*	*Cas9*	*Knockout*	[152]
Long shelf life	*ALC/alc & alc/alc*	*FJ404469*	*Agrobacterium*	*Cas9*	*Knockin*	[153]
Plant architecture, fruit size & inflorescence branching,	*WUS* & *CLV3*	*M88, S. pimpinellifolium*	*Agrobacterium*	*Cas9*	*Knockout*	[154]
Lycopene-1, Lycopene-3 & Lycopene-5	*G3P*, *DXS*, *CGPPS*, *PDS*, *ZISO*	*Alisa Craig*	*Agrobacterium tumefaciens mediated*	*Cas9*	*Knockout*	[140]
Carotenoid metabolism	*Psy1* & *CRTISO*	*Micro-Tom*	*Agrobacterium*	*Cas9*	*Knockin/geminiviral replicon*	[144]
γ-aminobutyric acid metabolism	*CAT9*, *GABA-TP1,2,3* & *SSADH*	*Alisa Craig, Micro-Tom*	*Agrobacterium*	*Cas9*	*Knockout*	[141]
Carotenoid synthesis	*Psy1 & CrtR-b2*	*Red Setter*	*Agrobacterium*	*Cas9/sgRNA*	*Knockout*	[144]
Fruit colour (Pink)	*MYB12*	*Inbred lines*	*Agrobacterium*	*Cas9*	*Knockout*	[155]
Fruit ripening	*lncRNA1459*	-	*Agrobacterium*	*Cas9*	*Knockout*	[141]
DNA methylation	*DDM1a*, *b*	-	-	-	-	[156]
Fruit size, number, shape, plant architecture & nutrient content	*FW2.2*, *SP*, *FAS*, *CyCb, MULT* & *OVUTE*	*Solanum pimpinellifolium*	*Agrobacterium tumefaciens LBA4404*	*Cas9*	*Knockout*	[153]
Pollen viability	*SIMAPK20*	*Micro-Tom*	*Agrobacterium tumefaciens*	Cas9	*Knockout*	[154]
Leaf senescence	*SBPASE*	-	-	*Cas9*	-	[157]
Cell wall gene	*PL* & *PG*	-	-	-	-	[156]
Fruit ripening & development	*AP2a, FUL1, FUL2* & *NOR*	*Moneyberg, Alisa Craig*	*Agrobacterium tumefaciens*	*Cas9*	*Knockout*	[158]
Fruit development & ethylene response	*ACS2, ERFE1, ARF2B, ACS4, EIN2* & *GRAS8*	-	*Agrobacterium tumefaciens*	*pHNCas9 & pHNCas9HT*	*-Knockout*	[159]
Dwarfism & gibberellin response	*PROCERA*	*Moneymaker*	*Agrobacterium*	Cas9	*Knockout*	[160]
Pectin degradation	*PL, PG2a & TBG4*	*Alisa Craig*	*Agrobacterium (EHA105)*	*Cas9/sgRNA*	*Golden Gate cloning method*	[161]
Interspecific tomato hybrid	*RECQ4*	*S. lycopersicum (Money Maker), S. pimpinellifolium (G1.1554)*	*Agrobacterium (AGL1)*	Cas9	*Knockout*	[106]
Firmness & fruit colour	*TBG, PG2a* & *PL*	*Alisa Craig*	*Agrobacterium tumefaciens*	*Cas9/sgRNA*	*-Knockout*	[157]
Anthocyanin biosynthesis	*SIAN2*	*Indigo Rose*	*Agrobacterium*	Cas9	*Knockout*	[142]
Lycopene & carotenoids	*DDBI, DETI, CYC-B*	*Micro-Tom*	Agrobacterium	Cas9	*Target AID base editing*	[162]
Fruit flavour	*FLORAL4*	*F6 (RILs)*	Agrobacterium	Cas9	*Knockout*	[162]
Male sterility	*SlMS10*	*KS-13*	Agrobacterium	Cas9	*Knockout*	[146]
Fruit size, locule number	*ENO*	*S. pimpinellifolium*	Agrobacterium	Cas9	*Knockout*	[162]
Induced somatic recombination’s	*CRTISO locus*	*Micro-Tom*	*Agrobacterium tumefaciens (GV3101)*	Cas9	*-Knockout*	[163]
Plant generation	*AGO7*	*Micro-Tom*	Protoplasts (PEG)	Cas9	*CBEs/ABEs*	[164]
Plant gene targeting	*SlANT1, SlHKT1;2, SlEPSPS1*	-	*Agrobacterium*	*Cas12a*	***Knockin***	[165]
Male sterility	*Ms10^35^, GSTAA*	*Micro-Tom*	Agrobacterium	Cas9	*Knockout*	[166]
Fruit ripening	*NAM1*	*Alisa Craig*	Agrobacterium	Cas9	*Knockout*	[167]
Carotenoids	*CRTISO*	*PKM1 (Periyakulam 1)*	Agrobacterium	Cas9	*Knockout*	[168]
Male sterility	*AMS*	*Alisa Craig*	Agrobacterium	Cas9	*Knockout*	[169]
Male sterility	*CMT4*	*Alisa Craig*	Agrobacterium	Cas9	*Knockout*	[170]
Fruit softening	*PG*	*Micro Tom*		Cas9	*Knockout*	[171]
Plant growth habit	*SP, SP5G*	*Red Setter*	Protoplasts (PEG)	Cas9	*Knockin/RNPs*	[172]
Plant regeneration	*SGS3, PR-1, RDR6, Mlo1, ProSys*	*S. peruvianum*	Protoplasts (PEG)	Cas9	*Knockin/RNPs*	[173]
Fruit ripening	*MIR164A (TARGETS: NAM2 &3)*	*Micro-Tom*	Agrobacterium	Cas9	*Knockout*	[174]
Plant regeneration	*SP, SP5G*	*Red Setter, Ailsa Craig, M82, Moneymaker*	Protoplasts (PEG-Ca^2=^)	Cas9	*RNPs*	[172]
Male Sterility	*RBOH, RBOHE*	*Alisa Craig*	Agrobacterium	Cas9	*Knockout*	[175]
Fruit number & weight	*KLUH (CYP78AFA family)*	*S. pimpinellifolium*	Agrobacterium	Cas9	*Knockout*	[176]
Fruit size	*BZR 1.7, SUN*	*Alisa Craig*	Agrobacterium	Cas9	*Knockout*	[177]
Chlorophyll, *B. cinerea*, Carotenoid	*GF*	*Moneymaker, San Marzano*	Agrobacterium	Cas9	*Knockout*	[178]
Recoloring red-brown fruits and high chlorophyll levels	*VIGE of STAYGREEN 1 (SGR1)*	*Micro-Tom*	Agrobacterium	Cas9	*Editing produced indel*	[64]
Induction of parthenocarpy traits	*SlANT1, SIAA9*	*ET5, ET 8 (Elite tomato)*	Agrobacterium	Cas9	*Overexpression and Knockout*	[179]
Fruit ripening	*SlSAHH2*	*Wild type*	Methylation mechanism	Cas9	*Knock out*	[180]
GABA accumulation	*SIGAD2 & SIGAD3*	*K19*	*Agrobacterium tumefaciens*	Cas9	*Knock out*	[181]
Male sterility	*MS1 & AMS-1*	*Micro-Tom*	Agrobacterium	Cas9	*Knock out*	[182]

**Table 4:** CRISPR/CAS-9 in potato for insect, disease resistance, and self-incompatibility.

Targeted Traits	Targeted Genes	Genotypes	Transformation System	CRISPR/Cas System	Knockin/ Knockout	References
**Insect, disease resistance**						
Potato mosaic virus-Y	*Cas13a/sgRNA*	*Desiree*	*Agrobacterium*	*LshCas13a*	*Transgenic plant developemtn*	[183]
Virus resistance, osmotic stress	*Coilin*	*Chicago*	*Apical meristem cell*	*Cas9*	*-Knockout*	[36]
*Leptinotarsa decemlineata*	*vest (vestigial gene)*	*-*	*-*	*Cas9/RNAi*	*Knockout*	[184]
**Potato mosaic virus-X**	*(sg)RNA*	*-*	*Agrobacterium*	*Cas9*	*Knockout*	[185]
Late blight resistance	*StDND1*, *StCHL1, StDMR6-1, StDMR6-2*	*Desiree*	*Agrobacterium*	*Cas9*	*Knockout*	[149]
Herbicidal tolerance	*StALS1 & StALS2*	*Desiree*	*Agrobacterium*	*Cas9/CBE (cytidine base editing)*	*Base editing*	[186]
Virus-induced genome editing Potato mosaic virus-X	*NbFT, NbPDS3, NbXT2B*	*Solanaceous plants (Nicotinana benthamina/potato*	*Agrobacterium*	*Cas9*	*Knockout*	[187]
Improvement in cold tolerance	*Vinv*	*Solanum lycopersicum* L.	*Agrobacterium mediated*	Cas9	*Knockout*	[188]
Resistance against multiple diseases	*Stdmr6-1*	*Solanum lycopersicum* L.	*Agrobacterium mediated*	Cas9	*Knockout*	[189]
**Self-incompatibility/compatibility**						
Self-incompatibility	*S-RNase*	*S. tuberosum Gp. Phureja (S15-65)*	*Agrobacterium*	*Cas9*	*Knock out*	[190]
Self-incompatibility	*S-RNase*	*DRH-195, DRH 310 F1*	*Agrobacterium*	*Cas9*	*Knock out*	[148]
Self-compatibility	*Sli*	*Solanum lycopersicum × S. habrochaites*	*Agrobacterium*	*Cas9*	*Knock out*	[147]

**Table 5:** CRISPR/CAS-9 in potato for quality improvement.

Targeted Traits	Targeted Genes	Genotypes	Transformation System	CRISPR/Cas System	Knock in/Knockout	References
Altered starch quality	*GBSS*	Kuras	*Protoplasts (PEG)*	*Cas9*	*Knockout*	[191]
Altered starch quality	*GBSS*	Kuras	*Protoplasts*	*Cas9/RNP*	*Knockout*	[192]
Altered starch quality	*GBSS*	Desiree & Wotan	*Protoplasts*	*Cas9*	*Knockout*	[197]
Altered starch quality	*GBSSI*	Sayaka	*Agrobacterium*	*Cas9*	*Knockout*	[193]
Altered starch quality	*GBSSI*	Desiree	*Agrobacterium*	*Cas9*	*Knockout*	[186]
α-solanine-free hairy roots	*St16DOX gene*	*May Queen*	*A. Rhizogenes*	*Cas9*	*Knockout*	[194]
Enzymatic browning Polyphenol Oxidase	*StPPO2*	Desiree	*Protoplasts*	*Cas9/RNP*	*Knockout*	[195]
Frameshift mutations & *S*. *aureus*-cytosine base editor	*Staphylococcus aureus (SaCas9)*	Desiree	*Agrobacterium*	*Cas9*	*Knockout-*	[81]
Amylose starch	*Sbe1 & Sbe2*	Desiree	*Protoplasts*	*Cas9/RNP*	*Knockout*	[196]
Steroidal Glycoalkaloids	*StSSR2*	*Atlantic*	*Agrobacterium*	*Cas9*	*Knockout*	[198]
Starch branching enzymes	*StSBE3*	*Sayaka*	*Agrobacterium tumifaciens*	*dMac3-Cas9*	*Knockout*	[198]
Starch quality	*SBE1, SBE2*	Desiree	*Agrobacterium & protoplasts (PEG)*	*Cas9*	*Knockout*	[195]
Starch synthesis	*StSS6 (Starch synthase gene)*	Desiree	*Agrobacterium*	*Cas9*	*Knockout*	[199]
Carotenoid biosynthesis	*PHYTOENE desaturase (PDS)*	Desiree	*Agrobacterium*	*Cas9*	*Knock in*	[200]
Carotenoid biosynthesis	*StPDS*	DMF (diploid, self-compatible F1hybrid	*Agrobacterium rhizogenes*	*Cas9*	*Knockout*	[201]
Carotenoid biosynthesis	PDS & Coilin	*Chicago*	*In Vitro*	*Cas9*	In vitro *evaluation*	[202]

**Table 6:** CRISPR/CAS-9 in brinjal (*Solanum melongena* L.) for disease resistance and other aspects.

Targeted Traits	Targeted Genes/Locus/Site	Genotypes	Transformation System	CRISPR/Cas System	Knock in/Knockout	References
Antifungal resistance	*DMR1, DMR6, EDR1, PMR4,5,6*	*Solanum melongena*	*gRNA design*	*Cas9*	*Identification of target loci*	[208]
Fruit flesh browning	*PPO gene (SmelPPO4, SmelPPO5, and SmelPPO6)*	*Black beauty*	*Agrobacterium-mediated transformation*	*hCas9*	*Knockout*	[206]
*Phytophthora* spp.	*DMR6–1*	*Black beauty*	*Agrobacterium-mediated*	*Cas9*	*Knockout*	[209]
Eye colour alternation	*Tryptophan 2,3-dioxygenase (TDO) gene*	*Eggplant shoot and fruit borer*	*Microinjection*	*Cas9*	*Knockout*	[207]
Reduction of fruit browning	*PPO2 (Polyphenol oxidase 2)*	*Pusa purple long*	*Agrobacterium-mediated transformation*	*Cas9*	*Knockout*	[210]
Disruption of carotenoid biosynthesis	*SmPDS (phytoene desaturase)*	*Eggplant (inbred line)*	*Agrobacterium-mediated transformation*	*Cas9*	*Knockout*	[211]

**Table 7:** CRISPR/CAS-9 in *Capsicum* spp. for disease resistance and other aspects.

Targeted Traits	Targeted Genes/Locus/Site	Genotypes	Transformation System	CRISPR/Cas System	Knock in/Knockout	References
Highly specific CRISPR/Cas9 editing sites	*NGG-PAM sites*	*Zunla-1’*	*-Stie editing*	*Cas9*	*-Site editing*	[215]
DNA Free screening	*CaMLO2*	*CM334, Dempsey*	*protoplasts*	CRISPR/RNPs (Cas9/Cas12a	*Knockin*	[213]
Anthracnose resistance	*CaERF28*	*Arka Lohit*	*Agrobacterium tumefaciens*	*CAS9/sgRNA*	*Knockout*	[214]
Stable transformation method	*CaMLO2*	*CM334, Dempsey*	*Agrobacterium tumefaciens*	In vitro *(tissue culture)*	*Knockout*	[212]
Biosynthesis of carotenoids	*CaPDS (Phytoene desaturase)*	*G4*	*Biolistic (particle bombardment)*	*Cas9*	*Knock out*	[216]
Disease resistance	*CaMLO2*	*D21101, D21102,E21301, E21302, F21201,J21401*	*Protoplast and PEG-mediated delivery*	*Cas9*	*Knock out*	[48]
Male sterility	*CaPIP5K4-1*	*Capsicum annuum* L.	*Virus-induced gene silencing*	*Cas9*	*Silencing/knock down*	[217]
Parthenocarpy induction	*CaPAD1*	*Dempsey, Younggo, C15*	*Leaf-derived protoplast*	*Cas9-RNP*	*Knockout*	[218]
Concave v/s pointed fruit tip regulation	*CaPCR1*	*LRS, SBS*	*qRT-PCR*	*Cas9*	*Mapping and gene expression analysis*	[219]
Stem strength	*CaSLR1*	*Capsicum annuum* L. *(Pepper)*	*Yeast hybrid*	*Cas9*	*Knock down*	[220]
Virus-induced gene editing	*CaPDS, FASCICULATE*	*TG-S#1 (transgenic line)*	*TRV (Tobacco rattle virus 2 (TRV2) based vectos*	*Cas9*	*Knock out*	[221]
Antifungal resistance	*DMR, EDR*	*Sweet pepper*	*Insilico-gRNA design*	*Cas9 g-RNA*	*Knockout*	[222]

## Data Availability

No new data were created or analyzed in this study. Data sharing is not applicable to this article.
